# De novo Neurosteroidogenesis in Human Microglia: Involvement of the 18 kDa Translocator Protein

**DOI:** 10.3390/ijms22063115

**Published:** 2021-03-18

**Authors:** Lorenzo Germelli, Eleonora Da Pozzo, Chiara Giacomelli, Chiara Tremolanti, Laura Marchetti, Christian H. Wetzel, Elisabetta Barresi, Sabrina Taliani, Federico Da Settimo, Claudia Martini, Barbara Costa

**Affiliations:** 1 Department of Pharmacy, University of Pisa, 56126 Pisa, Italy; lorenzo.germelli@phd.unipi.it (L.G.); eleonora.dapozzo@unipi.it (E.D.P.); chiara.giacomelli@unipi.it (C.G.); chiara.tremolanti@phd.unipi.it (C.T.); laura.marchetti@unipi.it (L.M.); elisabetta.barresi@unipi.it (E.B.); sabrina.taliani@unipi.it (S.T.); federico.dasettimo@unipi.it (F.D.S.); barbara.costa@unipi.it (B.C.); 2 Department of Psychiatry and Psychotherapy, Molecular Neurosciences, University of Regensburg, 93053 Regensburg, Germany; christian.wetzel@ukr.de

**Keywords:** 18 kDa TSPO, human microglial cells, neurosteroidogenesis, neurosteroids, CYP11A1, StAR protein, BDNF, TSPO ligands

## Abstract

Neuroactive steroids are potent modulators of microglial functions and are capable of counteracting their excessive reactivity. This action has mainly been ascribed to neuroactive steroids released from other sources, as microglia have been defined unable to produce neurosteroids de novo. Unexpectedly, immortalized murine microglia recently exhibited this de novo biosynthesis; herein, de novo neurosteroidogenesis was characterized in immortalized human microglia. The results demonstrated that C20 and HMC3 microglial cells constitutively express members of the neurosteroidogenesis multiprotein machinery—in particular, the transduceosome members StAR and TSPO, and the enzyme CYP11A1. Moreover, both cell lines produce pregnenolone and transcriptionally express the enzymes involved in neurosteroidogenesis. The high TSPO expression levels observed in microglia prompted us to assess its role in de novo neurosteroidogenesis. TSPO siRNA and TSPO synthetic ligand treatments were used to reduce and prompt TSPO function, respectively. The TSPO expression downregulation compromised the de novo neurosteroidogenesis and led to an increase in StAR expression, probably as a compensatory mechanism. The pharmacological TSPO stimulation the de novo neurosteroidogenesis improved in turn the neurosteroid-mediated release of Brain-Derived Neurotrophic Factor. In conclusion, these results demonstrated that de novo neurosteroidogenesis occurs in human microglia, unravelling a new mechanism potentially useful for future therapeutic purposes.

## 1. Introduction

Microglia are specialized macrophages resident in the central nervous system (CNS). In addition to immune-related activities, microglia appear to carry out a wider spectrum of activities aimed at the physiological development and normal homeostasis of the mature CNS, including plasticity, adult neurogenesis, and cognitive processes [1,2,3]. The dysregulations of microglial activities have been proposed to promote the onset and progression of several pathological CNS conditions [2,4,5]. At steady state, microglia exhibit a resting phenotype, morphologically characterized by the presence of widely branched processes [4]. These are used by the microglia to carry out continuous surveillance of their surrounding areas. The microglia sense infection and injury and rapidly change from the resting phenotype to an activated one that is characterized by an “amoeboid” morphology [4]. This activation is a complex process characterized by the release of many pro-inflammatory mediators that contribute to the management of inflammatory processes and elimination of the pathogen (M1 phenotype) [4]. However, microglial excessive activation can cause pathological forms of neuroinflammation, contributing to the progression of neurodegenerative and tumor diseases. On the other hand, it has been proposed that the resolving phase of the pathological condition is solicited by the presence of the alternative form of activated microglia (M2 phenotype), able to release various anti-inflammatory factors, including molecules responsible for tissue repair [4,6,7]. Molecular mechanisms that regulate the transition of microglia from resting to the activated states are currently extensively debated and still poorly understood. Understanding the mechanisms underlying the modulation of these phenotypes is important in deciphering the specialized functions and exploiting their therapeutic potential [8,9]. Remarkably, what we know about microglia comes mainly from data obtained from mouse models both in vitro and in vivo, while little is known about human microglia.

The 18 kDa translocator protein (TSPO) is largely expressed in both murine and human microglia [10,11,12,13,14]; therefore, its ability to modulate microglial function and dysfunction has been considered. Notably, TSPO plays a key role in modulating inflammation, and it seems to be particularly overexpressed in neurodegenerative contexts, in which the inflammatory component stands out [11,15]. Over the years, many ligands targeting this protein have been identified and proposed for the treatment of different CNS pathologies, by virtue of their potential to improve the cellular processes promoted by TSPO functions [16,17,18]. Among these, neurosteroidogenesis seems to be the most peculiar, and the most debated one [19,20,21]. Since neurosteroidogenesis as a whole is a conserved process, its mechanism is known, albeit with some species-specific differences [22]. Neurosteroidogenesis is mediated by two mitochondrial multiprotein complexes: the transduceosome and the metabolon [20,23]. The scheme comprehensive of the currently proposed members of transduceosome and metabolon can be found in our recent review [23]. The transduceosome is responsible for the transport of free cholesterol from cytoplasm to mitochondrion surface and its transfer from the outer mitochondrial membrane (OMM) to the inner one (IMM). Even if the mechanistic details underlying the transduceosome have not been fully clarified, the StAR protein seems to move cholesterol through the OMM, while TSPO appears to regulate cholesterol translocation [23,24,25]. Following transduceosome activities, the 37 kDa StAR cytosolic precursor is processed into the 30 kDa cleaved form, a sign of successful cholesterol delivery [23,24,25]. Then, the steroidogenic metabolon, a multimeric protein complex spanning the OMM, is responsible for metabolism of cholesterol by cytochrome P450 family 11 subfamily A polypeptide 1 (CYP11A1) in the IMM. CYP11A1 is an enzyme able to convert cholesterol into pregnenolone, a reaction that is considered steroidogenesis’s rate limiting-step [20,23,25], as pregnenolone is the precursor to all neurosteroids.

Neurosteroids have been proposed as possible endogenous molecules able to contribute to the maintaining of physiological microglial activities [26,27]. Among the glial CNS component, astrocytes are the most active steroidogenic cells: in addition to the ability to synthesize pregnenolone, they can produce progesterone, dehydroepiandrosterone (DHEA), androstenedione, testosterone, and estradiol. In addition, oligodendrocytes are able to perform neurosteroidogenesis de novo, producing pregnenolone and androstenedione [22,28,29,30]. However, microglia were initially thought unable to locally produce de novo neurosteroids [31]. In fact, in basal conditions, primary cultures of murine microglia did not show gene expression for the enzyme of the rate-limiting step of steroidogenesis CYP11A1, or the enzymes involved in the metabolism of pregnenolone [31]. It was believed that microglia were able only to metabolize androgens and estrogens, using metabolites from other central and peripheral steroidogenic sources [31,32,33]. In contrast, very recent data highlighted the capacity of immortalized murine microglia to synthesize neurosteroids de novo [34]. Although little is known about the neurosteroidogenic activity of microglia yet, emerging evidence suggests that neurosteroid production could be important in maintaining CNS homeostasis and in resolving microglia-mediated inflammation [14,35].

It has been reported that neurosteroids can affect the release and/or functions of different neurotrophins [22]. Indeed, estrogen can regulate the expression of BDNF via the estrogen response element on the BDNF gene [36]; similarly, glucocorticoids are also involved in BDNF regulation [37]. In this context, it has been demonstrated that BDNF could be released by microglia to promote neuronal maturation, plasticity, and survival [38,39,40]. However, how the de novo neurosteroidogenesis could affect the release of a neurotrophic factor such as BDNF is still unclear.

In the present work, the de novo neurosteroidogenic capacity of human resting microglia was explored. To this end, two immortalized microglial clones were used as in vitro models: the embryonic-derived HMC3 clone, widely used by the scientific community, and the recently created adult-derived C20 clone [41,42]. Previous studies that aimed at characterizing these human clones have documented that they show the typical phenotypic and functional characteristics of normal microglia [41,42,43]. Here, pregnenolone production and the expression of the entire neurosteroidogenic pathway were evaluated in these microglial cell lines. Furthermore, in order to deepen the knowledge on potential peculiarities of the microglial neurosteroidogenic mechanism, an experimental strategy aimed at reducing or enhancing the function of TSPO was undertaken. Particular attention was paid to this protein, as it could have therapeutic potential for the control of the microglial reactive states and thus of related CNS disorders.

## 2. Results

### 2.1. Human Microglia C20 and HMC3 Cells Produce Pregnenolone De novo

**Figure 1:**
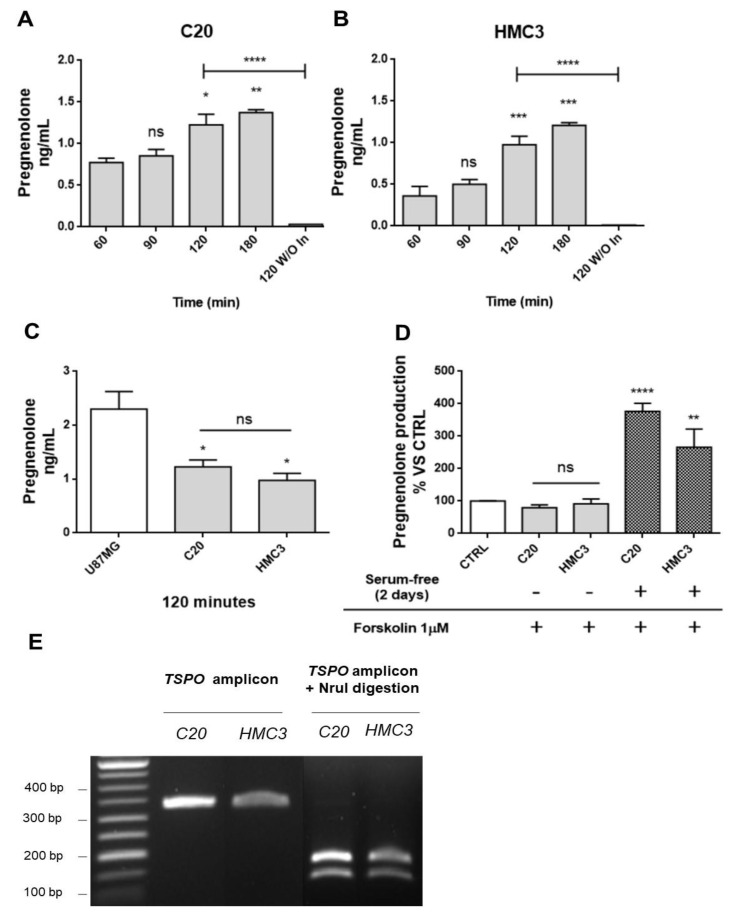
Human microglia C20 and HC3 cells: time-dependence of the pregnenolone production and genotyping for TSPO rs6971 polymorphism. (**A**,**B**) C20 and HMC3 cell samples were incubated in serum-free saline medium at time zero in the presence of the inhibitors trilostane and SU10603, and a kinetic analysis of pregnenolone released from microglia cells was performed. After various incubation times, the saline medium was collected from distinct cell samples and pregnenolone content was quantified by indirect ELISA. As shown in the figure, pregnenolone released from C20 and HMC3 cells increased in a time-dependent manner. A single incubation time (120 min) was conducted for the “inhibitor-free” samples named 120 without Inhibitors (120 W/O In). In this case, pregnenolone was not present in the collected samples, suggesting the conversion of pregnenolone into other neurosteroids. Pregnenolone levels were normalized based on the number of cells evaluated after crystal violet staining. Data are presented as means ± SEMs of three independent experiments. Statistical analysis was determined by one-way ANOVA followed by Bonferroni’s post-test: * *p* < 0.05, ** *p* < 0.01, *** *p* < 0.001 vs. 60 min; **** *p* < 0.0001, 120 W/O In vs. 120 min. (**C**) Comparison of pregnenolone production between human microglial and U87MG cells. (**D**) The classical stimulus of peripheral steroidogenesis (cAMP pathway activation) did not promote neurosteroidogenesis in microglial cells. Indeed, pregnenolone production by microglial cells was not stimulated following treatment with the known adenylate cyclase activator forskolin. However, the starvation phase before forskolin treatment led to a high increase in pregnenolone production. Data are represented as means ± SEMs of two independent experiments. The significance of the differences was determined by one-way ANOVA, which was followed by Bonferroni’s post-test: ** *p* < 0.01, **** *p* < 0.0001 vs. control. (**E**) The C20 and HMC3 cell genotyping for TSPO rs6971 was performed by restriction fragment length polymorphism (RFLP) analysis. The amplification product (329 bp) derived from genomic DNA was subjected to digestion by the restriction endonuclease NruI. Following the enzymatic digestion, the samples were subjected to agarose gel electrophoresis. Only the amplification product containing an Ala147 allele can be digested by NruI and generates restriction fragments (184 and 145 bp). As shown in the figure, C20 and HMC3 cells exhibited the restriction pattern typical of the Ala147 homozygous genotype.

As a first step, the ability of C20 and HMC3 cells to produce de novo steroids was investigated by evaluating the production of the first metabolite of neurosteroidogenesis, pregnenolone (Figure 1A,B). To quantify pregnenolone, its conversion to other metabolites of neurosteroidogenesis cascade was inhibited by the use of two synthetic compounds notoriously able to block the catalytic activity of specific steroidogenic enzymes. In particular, C20 and HMC3 were incubated in the presence of the following synthetic inhibitors: trilostane (inhibitor of 3β-hydroxysteroid dehydrogenase (3β-HSD)), which prevents the conversion of pregnenolone to progesterone, and SU-1060317 (inhibitor of 17α-hydroxylase/C17–20 lyase (P450c17 or CYP17A1)), which prevents the conversion of pregnenolone into DHEA. In parallel experiments, a known human CNS neurosteroidogenic model (U87MG tumor astrocytic cell line) was used as a positive control (Figure 1C) [44].

First, a kinetic measurement of pregnenolone released from C20 and HMC3 cells was performed. To that end, the complete culture medium was replaced with the saline medium (time 0). Then, the conditioned saline medium was collected at different times (time 60, 90, 120, or 180 min) and subjected to pregnenolone content quantification. The results showed similar kinetics for pregnenolone production in C20 and HMC3 cells: the release of the metabolite was augmented in a time-dependent manner, reaching a statistically significant increase after 120 and 180 min (Figure 1A,B). For inhibitor-free samples, the data showed that the C20 and HMC3 cell conditioned medium collected after a single incubation time (120 min) contained very low pregnenolone levels, below or slightly above the level of detection. Overall, these findings suggested that C20 and HMC3 cells produce pregnenolone de novo and can metabolize it to generate other products of the steroidogenic cascade. Similar amounts of pregnenolone were produced by C20 and HMC3 cells after 120 min (Figure 1A,B). Such quantities were in line with those previously documented for other murine central steroidogenic primary cell models, including microglia, using a series of experimental approaches [28,34,45,46,47]. As expected, the amount of pregnenolone observed for microglial cells was lower than the amount measured in the human astrocytic tumor cell line U87MG (Figure 1C).

To investigate whether the cAMP-mediated elective transduction pathway of peripheral steroidogenic sources could stimulate microglial de novo neurosteroidogenesis, C20 and HMC3 cells were exposed to the known adenylate cyclase activator forskolin. As shown in Figure 1D, forskolin treatment did not stimulate pregnenolone production in any of the microglial cell lines [47]. However, when the same treatment was applied to microglial cells, which were previously cultured in conditions that polarize them towards an activated phenotype [48], a strong increase in pregnenolone production was observed. This suggested that only activated microglial cells acquire the functional characteristics necessary to stimulate neurosteroidogenesis through the cAMP-mediated pathway.

The known TSPO single nucleotide polymorphism rs6971 has been functionally related to de novo steroidogenic efficiency [20,49]. This polymorphism substitutes Ala with Thr at amino acid position 147 (Ala147Thr), which is localized near the cholesterol binding CRAC domain. The TSPO polymorphism Ala147Thr has been documented to affect cholesterol binding and impair the rate of steroid synthesis [20,49]. Here, genotyping analysis revealed that C20 and HMC3 cells were homozygous for the common allele coding for Ala147, suggesting that both microglial clones exhibit the genotype associated with de novo steroidogenic efficiency (Figure 1E).

### 2.2. C20 and HMC3 Cells Express Transduceosome/Metabolon Key Proteins

The expression of proteins involved in the initial phases of neurosteroidogenesis was explored in C20 and HMC3 cells. Particular attention was paid to key members of the transduceosome and metabolon [20,23]; StAR and TSPO were chosen as transduceosome members, while CYP11A1 was chosen as the representative metabolon protein.

C20 and HMC3 cells were cultured in complete medium and analyzed for basal expression of the above-reported transduceosome and metabolon members by Western blot and immunofluorescence analyses.

**Figure 2:**
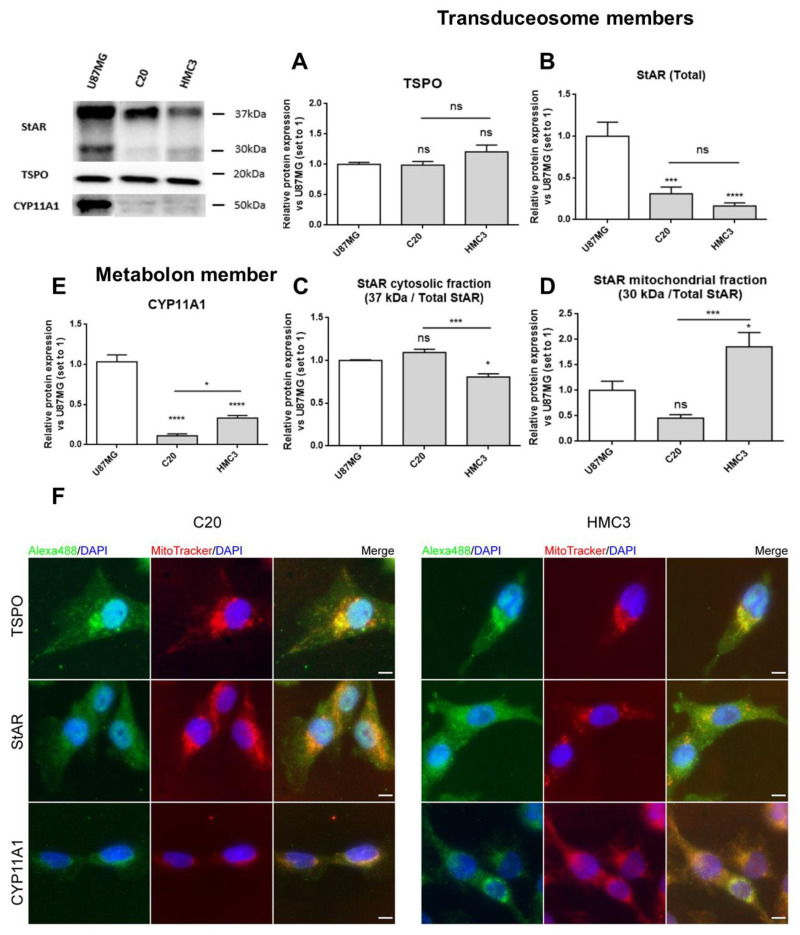
Expression of transduceosome/metabolon key proteins for neurosteroidogenesis in C20 and HMC3 cells. A representative Western blot panel for the expression of transduceosome members StAR and TSPO and the metabolon member CYP11A1 is reported. As shown in the panel, C20 and HMC3 cells expressed all transduceosome and metabolon members in basal conditions. (**A**–**E**) Densitometric analysis showed high expression of TSPO in both microglial cells (**A**), but StAR (**B**) and CYP11A1 (**E**) were expressed at lower levels if compared to the known neurosteroidogenic representative model (U87MG cells). Interestingly, the StAR cytosolic fraction (**C**) was reduced in HMC3 cells with respect to C20 cells. Moreover, the StAR mitochondrial fraction (**D**) and CYP11A1 in HMC3 cells seemed to be more expressed in comparison to C20 cells. Such differences could be due to the different developmental stages of the two microglial clones. Data are represented as means ± SEMs of three independent experiments. Statistical analysis was determined by one-way ANOVA followed by Bonferroni’s post-test: * *p* < 0.05, *** *p* < 0.001, **** *p* < 0.0001. (**F**) The expression of the transduceosome/metabolon members was also qualitatively evaluated with immunofluorescence analysis, which confirmed the Western blot results. The panel reports representative images of the co-localization study between TSPO, StAR, and CYP11A1 (green) with mitochondria stained with MitoTracker dye (red). DAPI staining is overlaid on both images to highlight cell nuclei. Scale bar: 10 μm.

The Western blot results demonstrated that all the studied neurosteroidogenic members were constitutively expressed in C20 and HMC3 cells, showing differences, above all, for StAR and CYP11A1 (Figure 2A–E). Both the microglial cells highly expressed TSPO (Figure 2A), suggesting that high levels of this protein may be required to maintain essential basal activities. Comparable levels of the total StAR (comprising 37 and 30 kDa forms) (Figure 2B) were observed in both microglial cells. However, reduced levels of the cytosolic 37 kDa StAR (Figure 2C) and increased levels of the mitochondrial 30 kDa StAR (Figure 2D) were observed in HMC3 than in C20 cells. Noteworthily, the presence of the StAR mitochondrial form pointed towards successful cholesterol delivery. CYP11A1 expression was higher in HMC3 than C20 cells.

The immunofluorescence analysis confirmed the presence of the investigated proteins and added details about their subcellular localization (Figure 2F). As expected, TSPO was detected mainly in the perinuclear region, as reported previously [47], and displayed good co-localization with mitochondria [50,51]. StAR appeared homogeneously distributed in cells, in agreement with its known dynamic shuttling between cytosol and mitochondria [20,23].The mitochondrial enzyme CYP11A1 presented perinuclear localization too, and the fluorescence intensity was higher in HMC3 than in C20 cells, in accordance with Western blot data (Figure 2E).

### 2.3. C20 and HMC3 Cells Showed Gene Expression of Key Steroidogenic Enzymes

**Figure 3:**
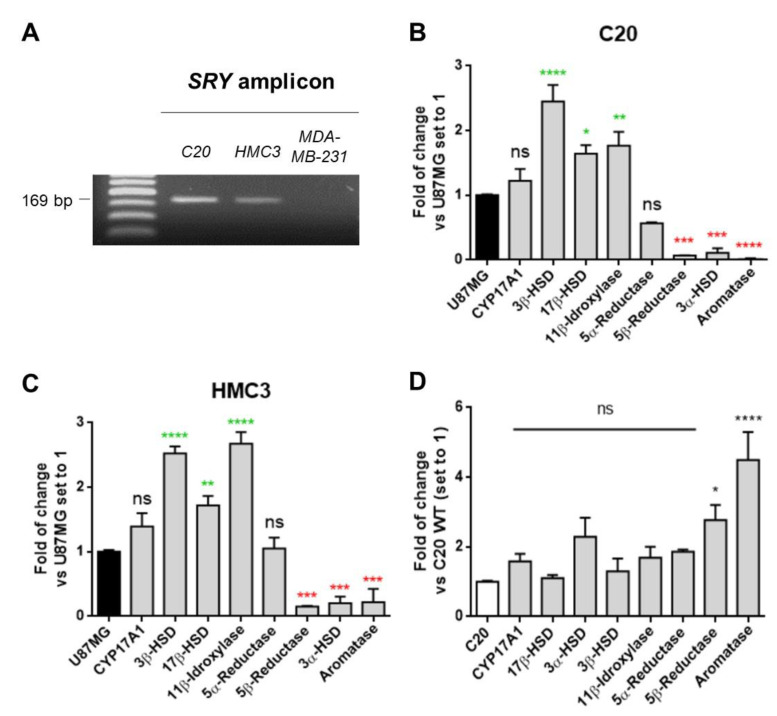
C20 and HMC3 cells: gendered origins and expression profiles of the enzymes involved in the neurosteroid biosynthetic pathway. (**A**) Results of genomic PCR evidenced the presence of SRY in both the microglial cells, suggesting their derivation from male individuals, whereas no amplicon was detected for the female-derived cell line MDA-MB.231. (**B**) and (**C**) The figures show the fold change analysis in mRNA levels of the enzymes involved in the neurosteroid biosynthetic pathway, comparing real-time RT-PCR data obtained from C20 and HMC3 cells to data of U87MG cells (set to 1). High mRNA levels for 17β–HSD, 3 β–HSD, and 11 β-hydroxylase; and very low mRNA levels for 5β-reductase, 3α-HSD, and aromatase were observed in C20 and HMC3 cells. The bars represent the means ± SEMs of three different experiments, and the significance of the differences was determined by one-way ANOVA, which was followed by Bonferroni’s post-test: * *p* < 0.05, ** *p* < 0.01, *** *p* < 0.001, **** *p* < 0.0001 vs. U87MG. (**D**) The mRNA data of the investigated enzymes were also compared between the two human microglial cell types, while setting C20 expression values to 1. No significant differences were evaluated for most of the enzymes, except for 5β-reductase and aromatase, which resulted in higher expression in HMC3 with respect to C20 cells. The significance of the differences was determined by one-way ANOVA, which was followed by Bonferroni’s post-test: * *p* < 0.05, **** *p* < 0.0001 vs. C20.

Given the gender-specific profiles of expression for steroidogenic enzymes [22], microglial cells were assessed for the known Y chromosome marker SRY (sex-determining region Y) [52] to rule out that the obtained results could be affected by a different genders among cells. The presence of SRY in C20 and HMC3 cells indicated that both clones were derived from male subjects, as has been reported for U87MG cells (ATCC Bioresource) (Figure 3A). The SRY amplicon was not detected in the MDA-MB-231 cell line, which was used as a negative control.

**Figure 4:**
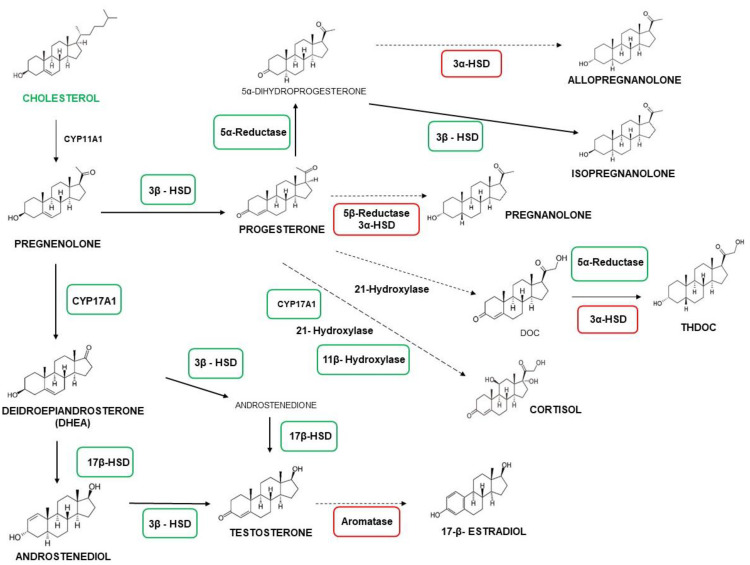
Neurosteroidogenesis pathway scheme. The biosynthetic pathway of neurosteroids is summarized. The enzymes that were transcriptionally expressed are in green boxes, except those at very low levels, which are in red boxes. A full arrow represents an enzymatic conversion that most probably occurred in the investigated microglial cells, whereas the dotted rows show a poorly represented one.

To investigate the enzymatic equipment of the neurosteroidogenesis cascade in human microglial cells, the gene expression of the key enzymes CYP17A1, 3β-HSD, 17β-HSD, 11β-hydroxylase, 5α-reductase, 5β-reductase, 3α-HSD, and aromatase was evaluated by real-time reverse transcriptase-polymerase chain reaction (real-time RT-PCR) analyses. The data obtained from the microglial cells were compared with those of the human astrocytic reference model U87MG. The microglial cells showed the expression of CYP17A1 and 3β-HSD mRNA coding for the enzymes that metabolize pregnenolone (Figure 3B,C), providing further evidence for the ability of these cells to produce neurosteroids de novo. In particular, compared to the astrocytic model, microglial cells showed similar and increased expressions of CYP17A1 and 3β-HSD, respectively (Figure 3B,C). In addition, the microglial cells expressed high levels of the enzymes 11β-hydroxylase and 17β-HSD, as previously reported for murine microglia models [35]. Within the 5α-/5β-reductase and the 3α-HSD systems responsible for the production of 3α,5α-reduced or 3α,5β-reduced derivatives of progesterone, testosterone, deoxycorticosterone (DOC), cortisol, and DHEA, C20 and HMC3 cells only expressed 5α-reductase mRNA at levels comparable to U87MG cells (Figure 3B,C). This is in accordance with previous data obtained in murine primary microglia [31]. Overall, these data suggested that, under basal conditions, microglial cells express low levels of those enzymes, which usually are upregulated under stress conditions [53]. Moreover, C20 and HMC3 cells displayed very low expression levels of aromatase, the enzyme responsible for the conversion of androgens into estrogen. This enzyme is known to be absent in mammalian CNS glial cells under basal conditions [29]. An overview of the neurosteroidogenic cascade is presented in Figure 4. The comparison between C20 and HMC3 cells showed a similar gene expression profile of steroidogenic enzymes, except for 5β-reductase and aromatase, which were higher in HMC3 than in C20 cells, even if the expressions of these genes were per se low compared to the reference control (Figure 3D).

### 2.4. TSPO Is Required to Maintain Basal De novo Neurosteroidogenesis in C20 and HMC3 Cells

In order to investigate the role of TSPO in de novo neurosteroidogenesis in human microglia, its expression was reduced by lentivirally-delivered, short hairpin RNA-mediated TSPO knockdown in C20 cells (C20 TSPO KD) [47]. Basal pregnenolone production was analyzed in C20 scramble (SCR) and TSPO KD cells.

**Figure 5:**
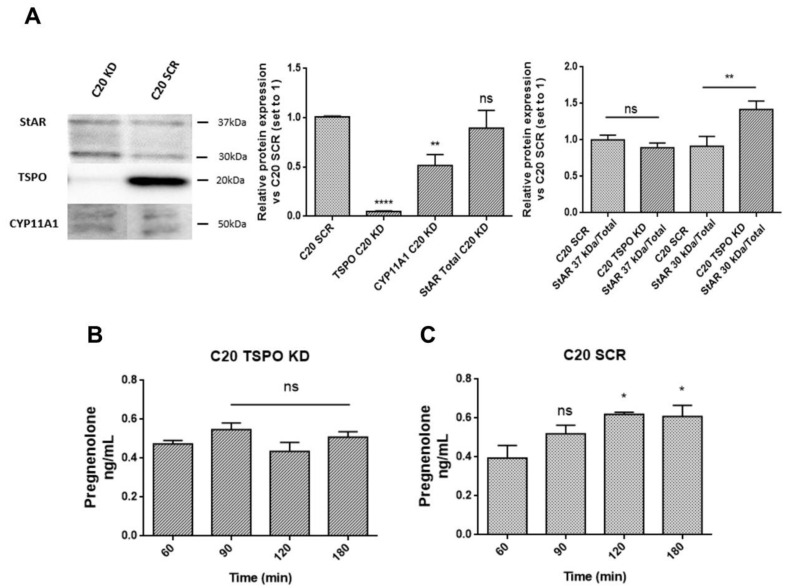
TSPO silencing reduced the steroidogenic ability of the microglial C20 cell line. (**A**) Representative figure of the transduceosome/metabolon protein expression (Western blot) and relative densitometric analysis in C20 TSPO KD and SCR control cells. In addition to the expected low level of TSPO, CYP11A1 expression turned out to be reduced in C20 TSPO KD cells; no differences were found in the total or cytosolic StAR expression. However, the mitochondrial form of StAR seemed to be more expressed as a result of a possible compensatory mechanism due to the TSPO knock-down. The bars show the outcomes of the densitometric analysis performed for three independent experiments; results are reported as fold changes ± SEMs of the protein expression vs. C20 SCR cells. The significance of the differences was determined by one-way ANOVA, which was followed by Bonferroni’s post-test: ** *p* < 0.01, **** *p* < 0.0001 vs. C20 SCR cells. (**B**,**C**) Kinetic analysis of pregnenolone production in C20 TSPO KD and SCR cells. The TSPO knock-down did not completely abolish the production of pregnenolone; however, no differences in its accumulation at the subsequent time points were observed. On the contrary, a statistically significant accumulation of pregnenolone after 120 and 180 min was observed in C20 SCR cells. Data are presented as means ± SEMs of three independent experiments. Statistical analysis was determined by one-way ANOVA followed by Bonferroni’s post-test: * *p* < 0.05 vs. 60 min.

First, TSPO expression was monitored in C20 TSPO KD cells to confirm the successful knockdown. Moreover, the levels of StAR and CYP11A1 expression were also examined (Figure 5A). The results showed strongly reduced TSPO expression in C20 TSPO KD compared to C20 SCR cells. Decreased expression of CYP11A1 was observed too (Figure 5A). This finding suggested impaired basal neurosteroidogenic activity of C20 TSPO KD cells. Interestingly, C20 TSPO KD cells showed no significant differences in the total and cytosolic StAR (37 kDa/Total StAR), but increased expression level of the 30 kDa mitochondrial form of StAR (30 kDa/Total StAR) compared to the SCR control cells. This data suggest a compensatory mechanism aimed at restoring normal transduceosome function, potentially compromised by the reduced TSPO expression.

The hypothesized impairment of the de novo neurosteroidogenic activity was corroborated by the lower amount of pregnenolone released from C20 TSPO KD cells compared with C20 SCR cells (Figure 5B,C). In C20 TSPO KD derived samples, pregnenolone levels did not reach statistical significance at any incubation time point, suggesting a lack of time-dependent accumulation. Therefore, the decreased basal neurosteroidogenic capacity, observed following TSPO silencing, indicated that microglia require high constitutive levels of TSPO to maintain this activity efficiently. ForC20 SCR cells, a time-dependent accumulation of pregnenolone was evidenced (Figure 5C). However, the observed pregnenolone levels in scramble C20 cells were lower than in wild-type C20 cells, suggesting that the genetic manipulation itself may affect the de novo neurosteroidogenic efficiency of C20 cells.

### 2.5. Potentiation of TSPO Function Increases Basal De novo Neurosteroidogenic Activity in C20 and HMC3 Cells

The role of TSPO in neurosteroidogenesis was further investigated by modulating its function with synthetic TSPO ligands in microglia cells. In particular, the ability of TSPO ligands to promote effectively the de novo production of neurosteroids in microglia was assessed. To this aim, synthetic TSPO ligands, which have been previously characterized for their steroidogenic activity in CNS cell models, were used [44,54,55,56]. In parallel, TSPO ligands with known high (XBD-173, PIGA1138 [57], and etifoxine) or poor (Ro5-4864, PK11195, and PIGA720) steroidogenic activity [54,55,56] were tested in C20 and HMC3 cells. The terms “poor/high steroidogenic activity” were referred to in a previous our paper, in which the parameter “residence time” (RT) was found to predict neurosteroidogenic efficacy of TSPO ligands [54,55,56]. TSPO ligands could be defined as “highly steroidogenic ligands” showing a maximum efficacy value of 250% or more (vs. control, set up at 100%), or “poorly steroidogenic ligands” showing a maximum efficacy value of 140−150% (vs. control, set up at 100%). Several studies have already suggested that the interaction of synthetic ligands with TSPO may promote the translocation of cholesterol into mitochondria for its cleavage by CYP11A1 [44,58,59].

**Figure 6:**
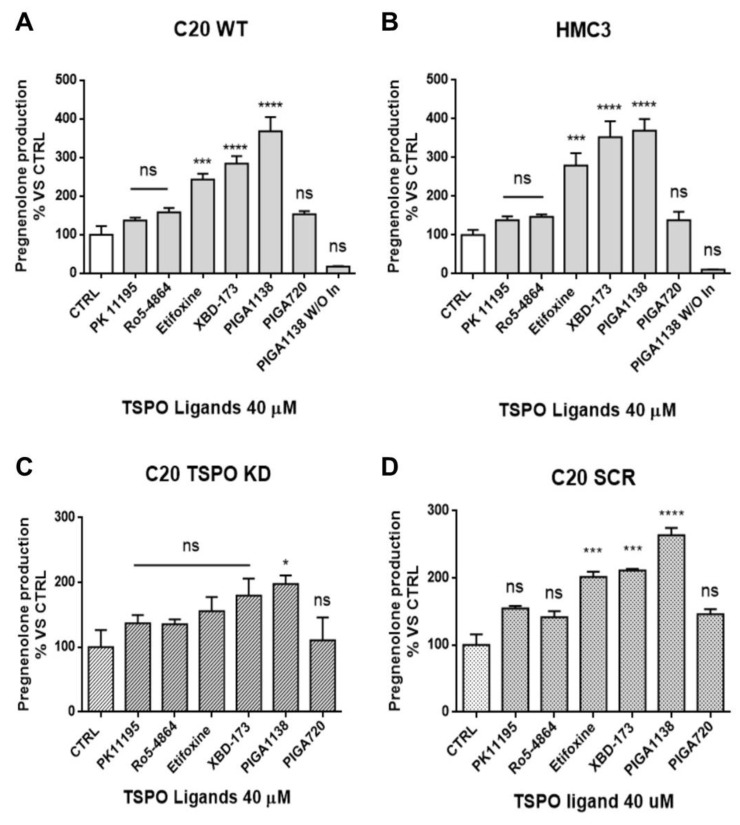
Pharmacological stimulation of neurosteroidogenesis by TSPO synthetic ligands in human microglial cells. (**A**,**B**) Cells were treated with TSPO ligands for two hours. After incubation, the saline medium was collected and used for the pregnenolone ELISA. The figure shows how TSPO ligands significantly promoted neurosteroidogenesis, particularly after the treatment with ligands characterized by high residence time (RT) at the TSPO binding site: etifoxine, XBD-173, and PIGA1138. The absence of pregnenolone metabolism inhibitors abolished pregnenolone accumulation in microglial cells treated with the most potent steroidogenic compound PIGA1138. (**C**) The TSPO knock-down reduced the TSPO ligands’ capacity to increase neurosteroidogenesis de novo, expect for PIGA1138. (**D**) TSPO ligands promoted de novo steroid production in C20 SCR control cells. Data are represented as means ± SEMs of independent experiments. The significance of the differences was determined by one-way ANOVA, which was followed by Bonferroni’s post-test: * *p* < 0.05, *** *p* < 0.001, **** *p* < 0.0001 vs. control.

The results showed that the TSPO ligands XBD-173, PIGA1138, and etifoxine stimulated pregnenolone production in C20 and HMC3 cells, confirming their high steroidogenic efficacy also in human microglial cells (Figure 6A,B). Importantly, these results suggested that pharmacological enhancement of TSPO function can stimulate de novo neurosteroidogenesis, probably because of increased cholesterol delivery into mitochondria. The lack of pregnenolone production stimulation by the known poorly steroidogenic TSPO ligands (PK11195 and Ro5-4864) suggested the specificity of the observed effect. In order to ascertain this aspect, the TSPO ligands were tested in C20 TSPO KD cells (Figure 6C). The results showed that the TSPO ligand neurosteroidogenic capacity was significantly reduced, except for the highly steroidogenic ligand PIGA1138 (Figure 6C). One characteristic that distinguishes PIGA1138 from the other tested highly neurosteroidogenic ligands (XBD173 and etifoxine) concerns its RT at the TSPO binding site. In particular, PIGA1138 remains bound to the protein for a longer time than the other ligands (RT values measured at PK11195 binding site: 141 min for PIGA1138, 127 min for XBD173, and 15 min for etifoxine) [54,55,56]. Probably, the stable interaction of PIGA1138 with residual amounts of TSPO could account for the neurosteroidogenic activity of this ligand observed in TSPO KD cells. C20 SCR control cells showed a similar TSPO ligand profile for pregnenolone production compared with C20 wild-type cells (Figure 6D), suggesting that genetic manipulation did not compromise the responsiveness of C20 cells to treatment with TSPO ligands.

### 2.6. Potentiation of TSPO Function Increases Brain-Derived Neurotrophic Factor (BDNF) Production in C20 and HMC3 Cells

In order to protect physiological brain functions, resting microglia cells regulate neurogenesis, neuronal survival and synaptic plasticity. It is well established that TSPO contributes to the maintaining of the resting phenotype of the microglia [14,60,61]; however, little is known about its effects on the release of neurotrophic and restorative factors. It has been already shown that neuroactive steroids, such as progesterone and allopregnanolone, could affect the BDNF release, in specific brain areas of mice [62,63]. In the present study, C20 and HMC3 cells were treated with the potent steroidogenic ligands XBD-173 and PIGA1138, and then the contents of BDNF and transforming growth factor-β (TGF-β) were measured in the conditioned medium.

**Figure 7:**
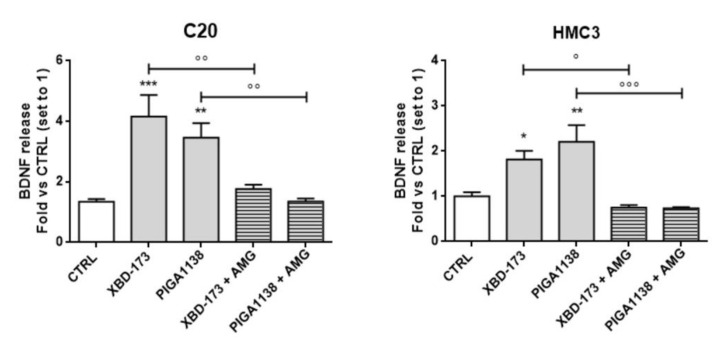
Effects of TSPO pharmacological stimulation on BDNF release from C20 and HMC3 cells. The microglial clones were treated with the potent steroidogenic TSPO ligands XBD-173 and PIGA1138 (100 nM) in serum-free medium for two hours. Then, the medium was replaced and the BDNF release was evaluated after 24 h. To evaluate the potential effect of neurosteroids on BDNF release, C20 and HMC3 cells were exposed to the inhibitor of CYP11A1 AMG (50 μM) for 1 h before TSPO ligand treatments. The collected medium was used for ELISA, and BDNF concentration was normalized with the number of cells. Data are presented as means ± SEMs of three independent experiments. As shown, the treatment of C20 and HMC3 with XBD-173 or PIGA1138 increased the BDNF production, and the effect was abolished by AMG pre-treatment. Statistical analysis was determined by one-way ANOVA followed by Bonferroni’s post-test: * *p* < 0.05, ** *p* < 0.01, *** *p* < 0.001 vs. control; ° *p* < 0.05, °° *p* < 0,01, °°° *p* < 0,001 vs. XBD-173 and PIGA1138.

Interestingly, both treatments significantly increased the ability of human microglial cells to release BDNF (Figure 7), while no differences were observed for the levels of TGF-β (data not shown). In order to investigate whether the BDNF release was dependent on neurosteroid synthesis, CYP11A1 activity was inhibited by pretreating the cells with aminoglutethimide (AMG), a CYP11A1 selective inhibitor. Interestingly, in AMG-pretreated samples, the effect of TSPO ligands on the BDNF release was significantly reduced, suggesting a dependence on neurosteroid activity. A previous study [64] reported that treating primary microglia cells with the selective TSPO ligand PK11195 led to increased release of BDNF in vitro. However, the results reported by Zhou et al. showed that PK11195 promotes BDNF release only at very high concentrations and for prolonged treatments (50 µM, 12 h). On the contrary, another group reported that PK11195 did not promote BDNF release in vivo [65]. This controversial effect on the BNDF release might be due to the short RT of PK11195, while a long RT is necessary for the production of significant steroid levels. Consistently, the combined high affinity and high RT of XBD-173 and PIGA1138 could favor efficient neurosteroidogenesis, which may directly have positive modulatory effects on microglial BDNF release, even at lower concentrations and reduced durations of treatment.

## 3. Discussion

Neuroactive steroids are the most potent endogenous modulators of microglial cells, and are capable of limiting their excessive reactivity, leading to beneficial effects in several neurodegenerative conditions. For several years there was widespread agreement that neurosteroidogenesis was not a specific function of microglial cells because in vitro and ex vivo models had evidenced gene transcription only for enzymes downstream the neurosteroidogenic cascade [31]. Thus, it has been commonly accepted that microglia lack de novo neurosteroidogenic capacity and have to be supplied with the first metabolites from other steroidogenic cell sources [31,32,33]. For this reason, the scientific community has paid poor attention to this biosynthetic process in microglia, while mainly investigating the effects induced by exogenously supplied neurosteroids [22]. This paradigm was questioned a few months ago following a study that has shown the ability of murine BV-2 microglial cells to produce de novo neurosteroids by the use of liquid chromatography with tandem mass spectrometry [34]. In the meantime, important results concerning this issue were obtained by our group in human microglia and reported in the present manuscript. Actually, little is known about human microglia, when also considering that substantial differences are emerging with respect to the murine-derived microglia [43,66]. Thus, it is of fundamental importance to deepen our knowledge about de novo neurosteroidogenesis in human microglia in order to draft a precise mechanistic model to direct new effective therapeutic interventions.

Herein, for the first time, the functional characterization of the neurosteroidogenic metabolic pathway was undertaken in two human microglial cell lines (C20 and HMC3), maintained in their resting condition. The results demonstrated that human microglia can produce and metabolize the first metabolite of neurosteroidogenesis, pregnenolone, suggesting that human microglia do have de novo neurosteroidogenic capacity. In line with this evidence, C20 and HMC3 cells constitutively expressed the key proteins of transduceosome/metabolon machinery that are notoriously required for the initiation of the biosynthetic process. Furthermore, they transcriptionally expressed the enzymes 3β−HSD and CYP17A1 that catalyze the conversion of pregnenolone to progesterone or DHEA, respectively.

The ability of human microglia to trigger neurosteroidogenesis indicated that the de novo production of neurosteroids appears to be required for the autocrine/paracrine regulation of the functionality of resting microglial cells. Indeed, starting from pregnenolone and progesterone, several other neurosteroids with peculiar effects can be produced [22]. Based on the cell type, different gene expression of the enzymes of the neurosteroidogenic cascade can direct the steroid metabolism. In both the investigated microglial clones, the enzymes 17β−HSD and 3β−HSD responsible for the production of androstenediol and progesterone were highly expressed. Conversely, both the microglial cells did not express the 5α-/5β-reductase, 3α-HSD, and aromatase enzymes, which are generally not required for activities under normal conditions. Notably, the androstenediol translation pathway has been implicated not only in the resolution of microglia-mediated inflammation, but also in the maintenance of normal CNS homeostasis by interacting with the ERβ receptor [35]. Progesterone is notoriously recognized to be a key steroid for regulating microglial activities involved in both the neuroinflammatory response and neuronal plasticity [67,68,69,70].

Several proteins are involved in steroid synthesis; among these, TSPO plays a pivotal role. TSPO binds cholesterol in coordination with the StAR protein, allowing the cholesterol translocation into mitochondria, which represents a crucial step of neurosteroidogenesis [20,23]. The high constitutive level of TSPO, observed in both the human microglial cells, suggested that this protein is important for maintaining the essential cellular activities. In resting human microglia, the crucial role of TSPO in mitochondrial metabolic activity has been recently documented [47]. Here, TSPO was found to have an important role in maintaining neurosteroidogenic efficiency in resting microglia. Indeed, following the TSPO silencing, reduced pregnenolone production was observed. However, the production of pregnenolone was not completely abolished, in line with previous findings [47].

In the past, results from studies involving TSPO knock-down or knock-out in vitro and in vivo models fueled doubts on TSPO involvement in the neurosteroidogenesis process [19,20]. However, with TSPO being highly conserved across all animal species and essential for cellular homeostasis [71,72], the possible establishment of compensatory mechanisms following TSPO silencing cannot be excluded. As reported in this work, silencing of this fundamental protein establishes a compensatory mechanism based on the increased expression of the mitochondrial StAR that probably compensates for the reduced cholesterol influx to the mitochondria due to the silencing of the TSPO gene. These data confirmed the results obtained by Fan et al., who observed increased expression of StAR protein in TSPO-deficient MA-10 cells [73]. The fact that the cells aim to restore this activity, when compromised, not only establishes the importance of TSPO in de novo neurosteroidogenesis, but also reinforces the key role of this metabolic pathway in maintaining the normal function of microglia. Noteworthily, the neurosteroidogenic efficiency allowed by the high levels of TSPO could be a pivotal requirement for the maintaining of the resting state. In support to this hypothesis, our previous results had shown that the TSPO silencing affects the microglial activation [14]. In particular, TSPO reduction has caused the imbalance of basally controlled production of anti-/pro-inflammatory cytokines favoring the release of the pro-inflammatory one. Future studies aimed at investigating the direct correlation of TSPO function with the increase of specific neurosteroids and the microglia activation state are certainly to be encouraged.

A result with potential therapeutic implications concerns the enhancement of neurosteroidogenic efficiency following the TSPO pharmacological stimulation that causes an increase in the cholesterol supply to CYP11A1. As previously reported, residence time (RT) is confirmed as a selective parameter for the prediction of the in vitro neurosteroidogenic efficacy of a synthetic TSPO ligand [54,55,56]. PIGA1138 [57] remains bound to TSPO for the longest time and demonstrated the highest pregnenolone production in C20 cells. Interestingly, some degree of PIGA1138 neurosteroidogenic capacity remained in the TSPO silenced C20 cells too. This effect could be explained by the long interaction of PIGA1138 with the residual amount of TSPO that remained following the silencing procedure. However, the possibility that the PIGA1138 effect was the consequence of its interaction with additional cellular targets involved in the neurosteroidogenesis modulation cannot be excluded. Precise experiments aimed at clarifying the events underlying such a phenomenon are certainly encouraged for future studies.

Noteworthily, the possibility to stimulate neurosteroidogenesis by TSPO ligands opens the way to deepening our knowledge on the precise contribution of neurosteroids directly produced by microglia in the autocrine regulation of homeostatic activities. From a therapeutic perspective, the results obtained offer the basis for considering de novo neurosteroidogenesis as a new strategic therapeutic target for the treatment of pathological conditions associated with dysregulations of reactive microglia [17]. Our previous results have already demonstrated the promising therapeutic potential of this target in the context of neuroinflammation. Indeed, highly steroidogenic TSPO ligands have induced the shift of the pro-inflammatory phenotype to the restorative one in human microglia. The phenotypic shift was abolished when the known inhibitor of CYP11A1 AMG was used [14,44].

The local autocrine and paracrine effects promoted by neurosteroids are broad and complex and could be classified as genomic and non-genomic [74]. The latter represent*s the most prominent ones due to the effects of neurosteroids on different receptors, in particular, GABA_A_ and NMDA receptors [75,76]. Regarding genomic effects of neurosteroids, they are usually referred to as the action on gene expression of cytokines, chemokines and restorative factors, including neurotrophins, due to their effects on intracellular receptors, such as the receptors for progesterone, estrogen, androgens, and pregnane X. This work demonstrates for the first time a possible autocrine and genomic effect on the BDNF release mediated by the production of neurosteroids. BDNF represents a key neurotrophic factor responsible for neuronal survival whose levels have been shown to be reduced in several CNS pathologies [77,78,79,80]. Notably, the increase of BDNF release was linked to the pharmacological activation of TSPO. In this context, the extension of the analysis to other microglial neurotrophins, whose release can be promoted by the stimulation of the TSPO steroidogenic function, is certainly to be encouraged.

In conclusion, the demonstration that microglia can produce de novo neurosteroids suggests an important role of neurosteroidogenesis in the autocrine/paracrine modulation of microglia. This newly-demonstrated activity could represent a possible neuroprotective and modulatory mechanism of the CNS microenvironment by human microglia. The altered levels of neurosteroids in several CNS pathologies involving neuroinflammation confirm the implication of these molecules in tissue homeostasis. In this light, the high expression of TSPO in human microglial cells and the possibility of stimulating its steroidogenic function suggests this protein as a promising therapeutic target both for the control of excessive microglial activation and for the restoration of physiological neurosteroids levels.

## 4. Materials and Methods

### 4.1. Materials

All materials for cell culture were purchased by Corning, New York, USA. All drugs and reagents used were purchased by Sigma-Aldrich (Saint Louis, MO, USA) except for PIGA1138 [57], PIGA720 [81], and SU10603 [82], which were synthesized as previously described.

### 4.2. Cell Culture

The human microglial HMC3 cell line was commercially available (ATCC, Manassas, Virginia) and were cultured in MEM (Minimum Essential Medium) containing 1.5 g/L sodium bicarbonate, non-essential amino acids (NEAA), L-glutamine, and sodium pyruvate, supplemented with 10% fetal bovine serum (FBS), 100 U/mL penicillin, and 0.1 mg/mL streptomycin. C20 human microglial cells were originally generated by David Alvarez-Carbonell et al. (Case Western Reserve University) [42]. C20 cells were cultured in DMEM-F12 medium supplemented with 10% of FBS, 100 U/mL penicillin, 0.1 mg/mL streptomycin, and neomycin (600 μg/mL) as a selector of the immortalized telomerase-expressing cells. C20 TSPO knockdown (KD) and scramble (SCR) cells were derived from stable transfection with lentiviral expression vector pLKO containing TSPO short hairpin RNA (shTSPO) or an SCR sequence [47]. C20 KD and SCR cells were cultured in the same medium of C20 cells, with the addition of blasticidin (2.5 μg/mL) for the selection of pLKO vector. U87MG cells, used as a positive control of neurosteroidogenic cells [44], were purchased from ATCC, and cultured in EMEM (Corning, New York, USA) supplemented with 10% FBS, 2 mM L-glutamine, 1% NEAA (Sigma-Aldrich), 100 U/mL penicillin, and 0.1 mg/mL streptomycin. All cell lines were cultured at 37 °C in 5% CO_2_.

### 4.3. Pregnenolone ELISA

Pregnenolone quantification was performed by ELISA, as previously reported [44]. Briefly, 10^4^ cells/well were seeded the day before in 96-multiwell plate, and kept in their culture media overnight. The day after, the culture medium was replaced at time zero with saline medium (140 mM NaCl, 5 mM KCl, 1.8 mM CaCl2, 1 mM MgSO4, 10 mM glucose, 10 mM (HEPES)–NaOH, pH 7.4, and 0.1% bovine serum albumin) containing the inhibitors of the further pregnenolone metabolism trilostane (25 μM) and SU10603 (10 μM). For the assessment of pregnenolone accumulation over time, the conditioned salt medium was collected at different time points (60, 90, 120, and 180 min), for a maximum of 3 h of incubation. For the evaluation of the effect of TSPO activity on pregnenolone release, cells were incubated in salt medium with the addition of TSPO ligands 40 µM or forskolin 1 µM for 2 h. For starved samples, cells were maintained in serum-free medium for 24 h, and then treated in the saline medium for 2 h as previously described. After collection, the medium was used for a competitive ELISA based assay for pregnenolone quantification (DB52031, IBL Internation, Hamburg, Germany). The absorbance intensity resulting from ELISA protocol was quantified by the Ensight Multimode Plate Reader instrument (PerkinElmer, Waltham, MA, USA) and then inversely related to the ng/mL of pregnenolone. Pregnenolone-quantified concentrations were normalized with the number of cells of each well by crystal violet staining. Briefly, cells were fixed with paraformaldehyde (PFA), diluted to 4% with phosphate buffer (PBS), for 15 min. After washes, 50 μL of a methanolic solution of crystal violet was added to each well and the plate was incubated for 30 min. Then, cells were washed and lysed by adding 100 µL of 1% SDS solution. The absorbance at 595 nm was read with the Ensight instrument.

### 4.4. Western Blot

Cells were seeded in P100 Petri Dishes at a density of 8 × 10^5^ cells. After 24 h, cells were lysed by adding RIPA buffer (0.5% sodium deoxycholate, PBS pH7.4, 1% Igepal, 0.1% SDS; and protease inhibitors—4 μg/mL apronitin, 1 μM ortovanoate, and 0.1 mg/mL PMSF). The cellular suspension was sonicated in ice and maintained on a rotating wheel at 4 °C for 2 h. Protein quantification of the cell lysates was performed by using the Bio-Rad DC Protein Assay, following the manufacturer’s protocol; 30 μg of each cell protein extract was mixed with the Laemmli solution and resolved by electrophoresis SDS-PAGE, supported by the use of precast stain-free gel (Bio-Rad, Hercules, California) composing of a gradient of polyacrylamide 4–20%. Then, the gel was activated by using ChemiDoc™ XRS·+ (Bio-Rad, California) instrument, and the separated proteins were transferred to PVDF membranes. Proteins were imaged to normalize the intensity of the investigated band with total protein [83]. The membrane-containing proteins were incubated with a suitable blocking solution for 2 h (5% non-fat dry milk or TBS, 5% BSA/0.1% Tween 20) and then overnight at 4 °C with a primary antibody. The day after, the membrane was washed with Tris-buffered saline (TBS) solution added with 0.05% Tween20 (Sigma-Aldrich). The membrane was incubated again for two hours at room temperature with anti-rabbit IgG light chains conjugated to peroxidase, washed with TBS-Tween, and then peroxidase was detected with the chemo-luminescent substrate ECL (Bio-Rad, California). Antibodies and dilutions used are listed below: Anti-TSPO Rabbit mAb (from Wetzel lab), 1:7500; anti-CYP11A1 Rabbit mAb (D8F4F, Cell Signalling Technology, Massachusetts, USA), 1:500; anti-StAR Rabbit mAb (D10H12, Cell Signalling Technology, Massachusetts, USA), 1:500; Anti-Rabbit IgG (A6154 Sigma Aldrich) 1:5000.

### 4.5. Genotyping of Human Microglial Cells for TSPO rs6971 Polymorphism

The microglial cell lines C20 and HMC3 were analyzed by Restriction Fragment Length Polymorphism (RFLP) assay to establish the presence of the human allelic variant rs6971 of TSPO, as previously reported [41]. Genomic DNA (gDNA) was extracted by using the “Genomic DNA extraction kit” (Thermo Fisher Scientific, Waltham, Massachusetts USA) from a cell pellet composed of 6 × 10^5^ cells approximately, following the manufacturer’s protocol. gDNA was quantified with NanoDrop Instrument (Thermo Fisher Scientific) and used as a template for PCR reaction performed with Phusion Master Mix (Thermo Fisher Scientific). Briefly, 50 ng of gDNA was mixed with DNA polymerase, HF buffer, and 500 nM of the specific primer pair (forward: TGGGACAGGCACTTGGGTGAAC; reverse: AAGCGTGACGGCCACCACATCA) to ensure the amplification of the region containing the investigated variant. The reaction was performed for 35 cycles following the thermal protocol: 98 °C for 30 s, 98 °C for 10 s, 68 °C for 20 s, 72 °C for 8 s, and a final extension phase at 72 °C for 7 min. The PCR mix was collected and subjected to overnight enzymatic digestion with NruI (Thermo Fisher Scientific). Digested and non-digested reactions were loaded on 2% agarose gels and submitted to electrophoresis. The bands of interest were visualized by using the ChemiDoc™ XRS·+ instrument.

### 4.6. Cell Sex Determination by SRY Gene PCR

The presence of the SRY gene was evaluated by PCR to assess the gender origin of the human microglial cells. The MDA-MB-231 cell line was used as a negative control due to the female gender origin. gDNA was extracted as previously described, and 50 ng was used for the PCR reaction using specific SRY primers pair (forward: CAGATCCCGCTTCGGTACTC; reverse: TTTGTCCAGTGGCTGTAGCG). The reaction was conducted with the same thermal protocol as Section 4.5, using an annealing temperature of 60 °C. PCR reaction products were loaded on 2% agarose gel for electrophoresis, and afterward the bands of interest were visualized by using ChemiDoc™ XRS·+ instrument.

### 4.7. Immunostaining of Transduceosome/Metabolon Protein

C20, HMC3 and U87MG cells were cultured on a round glass coverslips with a density of 3 × 10^4^ cells/well per 24-well plate. Cells were stained with addition of 500 nM MitoTracker^TM^ Red CM-H_2_Xros (ThermoFisher) diluted in cell medium for 45 min at 37 °C, before fixation with 4% of PFA for 15 min followed by three washes with PBS. Then the cells were permeabilized with a PBS solution containing 2.5% BSA and 0.3% Triton X100 for 10 min, blocked with the same solution without Triton for 1 h at room temperature, and then incubated overnight at 4 °C with primary antibody diluted in blocking solution (anti-TSPO 1:1000; anti-CYP11A1 1:100; anti-StAR 1:100). The day after, cells were washed with PBS, and incubated for 2 h with an anti-rabbit goat IgG conjugated with an Alexa Fluor 488 dye (Thermo Fisher Scientific, 1:100). After washes, cell nuclei were stained with DAPI (Sigma Aldrich) 1:5000 diluted in blocking solution. Coverslips were mounted with Vectashield (Vector Labs) on a microscope support and imaged with an epifluorescence microscope (Nikon E-Ri) using a 60X oil objective (NA 1.4). Sequential z-stacks in the blue, green, and red emission channels were acquired, respectively, for the detection of DAPI, TSPO/StAR/CYP11A11, and mitochondria at 60× magnification. Exposure time and acquisition parameters were kept constant for analysis of the green channel in the three different cell lines. The generated stacks were analyzed by using ImageJ program ImageJ (nih.gov). The z-projections of the stacks were background-subtracted, subjected to linear brightness/contrast enhancement, and merged for co-localization analyses.

### 4.8. Real-Time RT-PCR of Human Microglial Neurosteroidogenic Enzymes

Cells were seeded the day before the experiment in a six-well plate with a density of 2 × 10^5^ cells/well and maintained in complete culture media. Then, cells were detached by using Trypsin/EDTA and total RNA was extracted using the RNeasy Mini Kit (Qiagen, Hilden, Germany). The purity of the RNA samples was determined by measuring the absorbance ratio 260:280 nm, by using the NanoDrop Instrument (Invitrogen, Carlsbad, CA, USA). RNA (500 ng) was subjected to a retro transcription process performed by using the iScript cDNA Synthesis Kit (Bio-Rad, Hercules, CA, USA). Primers used for the RT-PCR reactions, listed in Table 1, were designed to specifically amplify regions bridging between two exons and to skip introns. The final RT-PCR reactions were performed with 50 ng of cDNA, 500 nM each of the forward and reverse primers, and 10 μL of SsoAdvanced Universal SYBR Green Supermix (Bio-Rad). All reactions were performed with the following temperature profiles: 95 °C for 15 s (enzyme activation), 98 °C for 30 s (initial denaturation), and suitable annealing and extension temperatures for 30 s. Denaturation, annealing, and extension steps were repeated for 40 cycles. The PCR specificity was determined using both a melting curve analysis and gel electrophoresis. Ct values were normalized on the beta-actin expression used as the housekeeping gene. The relative mRNA expression was calculated for each sample and gene by using the 2^-∆∆Ct^ method. In particular, the human microglial mRNA expression of the neurosteroidogenic enzymes was related to the one of the U87MG cell lines.

### 4.9. BDNF and TGF-β ELISA Quantification

C20 and HMC3 cells were seeded in a 24-well plate with a density of 10^5^ cells/well. The day after, cells were treated with 100 nM of TSPO ligands in a serum-free medium for 2 h, and then the medium was replaced with a fresh serum-free one and they were incubated for 22 h. In the end, the medium was collected, and centrifuged at 20,000× *g*, and 100 μL were used for the quantification of human BDNF and TGF-β levels by high sensitive ELISA kit (Cloud Clone Corp; BDNF: SEA011Mi, detection range 31.2–2000 pg/mL; TGF-β: SEA124 Hu, detection range 15.6–1000 pg/mL). To assess whether BDNF and TGF-β production was mediated by neurosteroids activities, cells were pre-treated with 50 μM of AMG, an inhibitor of the CYP11A1 enzyme, involved in the first phase of neurosteroidogenesis. Levels of BDNF and TGF-β were normalized on the number of cells by crystal violet staining and expressed as the fold changes of the untreated cells.

### 4.10. Statistical Analysis

The Graph-Pad Prism program (GraphPad Software Inc., San Diego, CA, USA) was used for data analysis and graphic presentation. All data are presented as the means ± SEMs. Statistical analysis were performed as indicated in each graphic. The *p*-value < 0.05 was considered to be statistically significant.

## Figures and Tables

**Table 1:** List of primers used for RT-PCR.

Gene	Primer Nucleotide Sequence	Anneal. Temp.	Product Size
CYP17A1	F: TAGGGGACATCTTTGGGGCTGGC R: CACTGATAGTTGGTGTGCGGCT	58 °C	150 bp
3α-HSD	F: GGTCACTTCATGCCTGTCCTGGG R: GCCAGTCCAACCTGCTCCTCA	65 °C	155 bp
3β-HSD	F: TGTGAAAGGTACCCAGCTCCTGT R: GGAGCGGGCCATGTGTTTTCC	55 °C	159 bp
17β-HSD	F: TGGGGCTGCCTTTCAATGACGT R: CGATCAGGCTCAAGTGGACCCCA	55 °C	111 bp
5α-Reductase	F: TTGGCTTGTGGTTAACGGGC R: AAGCCGCGCCTTGGACAGAC	58 °C	198 bp
5β-Reductase	F: GGTCCTCCAGCTAGATTATGTGG R: ACCATTTGCCATTCTCATCTCT	67 °C	101 bp
11β-hydroxylase	F: TCGGAACCCCAACGTGCAGCA R: GCACCAAGTCTGAGCTCGCCA	65 °C	182 bp
21-hydroxylase	F: AGGAGTTCTGTGAGCGCATGAG R: GGCAGGCATTAAGTTGTCGTCC	68 °C	137 bp
Aromatase	F: GCATGGCAAGCTCTCCTCATCA R: TCAACTCAGTGGCAAAGTCCA	65 °C	181 bp
β-actin	F: GCACTCTTCCAGCCTTCCTTCC R: GAGCCGCCGATCCACACG	58 °C	254 bp

## Data Availability

All data supporting the results are available in the manuscript.

## Abbreviations

TSPO	Translocator Protein 18 KDa
StAR	Steroidogenic Acute Regulatory protein
CYP11A1	Cytochrome P450 family 11 subfamily A member 1
BDNF	Brain-derived neurotrophic factor
TGF-β	Transforming Growth Factor β
KD	Knockdown
CNS	Central Nervous System
CRAC	Cholesterol Recognition Aminoacidic Consensus
DHEA	Dehydroepiandrosterone
CYP17A1	17α-hydroxylase/C17-20 lyase
3α-HSD	3α-hydroxysteroid dehydrogenase
shRNA	Short Hairpin RNA
SCR	Scramble
AMG	Aminoglutethimide
OMM	Outer Mitochondrial Membrane
IMM	Inner Mitochondrial Membrane
R	Residence Time
